# PTPN11 (SHP2) Is Indispensable for Growth Factors and Cytokine Signal Transduction During Bovine Oocyte Maturation and Blastocyst Development

**DOI:** 10.3390/cells8101272

**Published:** 2019-10-18

**Authors:** Muhammad Idrees, Lianguang Xu, Seok-Hwan Song, Myeong-Don Joo, Kyeong-Lim Lee, Tahir Muhammad, Marwa El Sheikh, Tabinda Sidrat, Il-Keun Kong

**Affiliations:** 1 Division of Applied Life Science (BK21 Plus), Gyeongsang National University, Jinju 52828, Gyeongnam Province, Korea; idrees1600@gmail.com (M.I.); xulianguang428@gmail.com (L.X.); siwd2002@gmail.com (S.-H.S.); jmd1441@gmail.com (M.-D.J.); mtahir.khan@gnu.ac.kr (T.M.); marwa.el-sheikh@hotmail.com (M.E.S.); tabindasidrat06@gmail.com (T.S.); 2 The King Kong Ltd., Daegu 43017, Korea; 0920-0728@hanmail.net; 3 Institute of Agriculture and Life Science, Gyeongsang National University, Jinju 52828, Gyeongnam Province, Korea

**Keywords:** SHP2 (*PTPN11*), PHPS1, cisplatin, growth factors, cytokines, MAP kinases, PI3K/AKT, blastocyst

## Abstract

This study was aimed to investigate the role of SHP2 (Src-homology-2-containing phosphotyrosine phosphatase) in intricate signaling networks invoked by bovine oocyte to achieve maturation and blastocyst development. *PTPN11* (Protein Tyrosine Phosphatase, non-receptor type 11) encoding protein SHP2, a positive transducer of RTKs (Receptor Tyrosine Kinases) and cytokine receptors, can play a significant role in bovine oocyte maturation and embryo development, but this phenomenon has not yet been explored. Here, we used different growth factors, cytokines, selective activator, and a specific inhibitor of SHP2 to ascertain its role in bovine oocyte developmental stages in vitro. We found that SHP2 became activated by growth factors and cytokines treatment and was highly involved in the activation of oocyte maturation and embryo development pathways. Activation of SHP2 triggered MAPK (mitogen-activated protein kinases) and PI3K/AKT (Phosphoinositide 3-kinase/Protein kinase B) signaling cascades, which is not only important for GVBD (germinal vesical breakdown) induction but also for maternal mRNA translation. Inhibition of phosphatase activity of SHP2 with PHPS1 (Phenylhydrazonopyrazolone sulfonate 1) reduced oocytes maturation as well as bovine blastocyst ICM (inner cell mass) volume. Supplementation of LIF (Leukemia Inhibitory Factor) to embryos showed an unconventional direct relation between p-SHP2 and p-STAT3 (Signal transducer and activator of transcription 3) for blastocyst ICM development. Other than growth factors and cytokines, cisplatin was used to activate SHP2. Cisplatin activated SHP2 modulate growth factors effect and combine treatment significantly enhanced quality and rate of developed blastocysts.

## 1. Introduction

An oocyte is a hub containing all of the information necessary for the successful nuclear and cytoplasmic maturation [1]. The process of maturation is initiated by triggering intricate signaling networks invoked by the oocyte to get competency for fertilization and blastocyst development. A variety of growth factors have been used to influence oocyte maturation and embryo development [2,3]. It has been widely accepted that facilitative growth factors and cytokines must be added to the medium due to their enormous effects on the oocyte in vitro maturation and embryo development [4,5]. Supplementation of these factors activate MAPK 3/1 signaling for oocyte maturation, and PI3K/p-AKT pathway for embryo development [5,6,7]. Previously, it was demonstrated that EGF-like growth factors an activator of EGF receptor not only regulate maternal mRNA translation through MAP kinases but also reinforced fertilized zygotes to attain developmental competency by activating the Ras/PI3K/AKT/mTOR pathway [8,9]. Similarly, insulin growth factor (IGF) [10] and fibroblast growth factor (FGF) were also reported to be involved in the induction of MAP kinase signaling during oocyte maturation and gap junction enhancement [2,11].

The status of protein tyrosine phosphorylation and dephosphorylation is central to growth factors, cytokines, and integrin-related signal transductions [12,13,14]. One of the important regulatory protein families that plays a major role during changes in the phosphorylation and dephosphorylation state of the protein is the Src family of phosphatases [15]. The *PTPN11* encoding protein SHP2 is ubiquitously expressed [16]. It is involved in the activation of several growth factor signaling cascades and plays a significant role in multifarious biological functions [16,17]. The response of SHP2 toward growth factors, hormones, and cytokines is due to its pronounced effect on the activation of the Ras (Retrovirus-associated DNA sequences)/MAPK cascade [18,19]. Phosphatases have to bind to their physiological substrates and EGF receptor (EGFR) was found to be a potent physiological substrate for SHP2 [20]. SHP2 phosphatase activity requires tyrosyl phosphorylation (Y542 and Y580) for MAP kinase pathway activation and also for PI3K signaling, as Y-phosphorylated SHP2 can form a tertiary complex with the scaffolding proteins Gab1/2 (Grb-associated-binding protein 1/2) and the p85 subunit of PI3K [21,22]. Previously it has been demonstrated that SHP2 dephosphorylate the EGF-R on its tyrosine 922, which is binding site for RAS/GAP. This dephosphorylation induce EGF signaling and resulting in promotion of RAS/MAPK activation [23]. Other than EGFR, SHP2 phosphatase activity is also important for FGF receptor signaling to activate MAP Kinases [24]. Moreover, SHP2 also interact with IGF [25], and LIF [26], for their signal transduction. Similarly, a wide range of literature is available regarding the role of SHP2 protein in the field of other cytokine signaling [27].

SHP2 catalytic activity is directly involved in the activation of many protein kinases expressed in oocyte and in cumulus cells, that control oocyte maturation and embryo development [28]. MAPK/ERK is well-known protein signaling cascade for oocyte maturation in many species and also play an important role in bovine oocyte maturation [29]. Activation of MAP Kinases regulates many protein targets in the cytoplasm and nucleus, which affects cell proliferation, nuclear membrane formation, chromatin condensation, microtubular reorganization, and the mode of expression of various genes, and SHP2 knockout or inhibition have direct effect on MAPK family [30,31]. In oocyte MAPK 3/1 cascade play pivotal role in meiotic cell cycle, by regulating maternal mRNA through phosphorylating and degrading CPEPB-1 (Cytoplasmic Polyadenylation Element Binding Protein-1) [6]. Other than MAPK, PI3K/AKT pathway also play significant role in GV breakdown and embryo development. SHP2 catalytic activity is required for the activation of PI3K/AKT signaling, which is abundantly expressed in bovine oocytes and play essential role in maturation and development [32,33,34].

SHP2 a core component of RTKs and cytokines signal transduction has never been explored at oocyte stage in any species. The current study was designed to investigate the expression of PTPN11 (SHP2) in bovine ovary, pre-ovulatory follicle, COCs (cumulus oocyte complexes), mature oocyte and embryo. We hypothesize that if SHP2 has been expressed in the bovine oocyte, then it will be an essential regulator for oocyte maturation and will play critical role in growth factors and cytokines signal transduction during embryo development and blastocyst implantation. SHP2 active role was analyzed by inhibiting it with its specific inhibitor PHPS1 [35] during different developmental stages. Furthermore, cisplatin (selective activator of SHP2) [36] alone and with growth factors was used to precisely understand the mechanism of SHP2 during bovine oocyte maturation and embryo development.

## 2. Material and Methods

All the chemicals and reagents were obtained from sigma-Aldrich (St. Louis, MO, USA), unless otherwise noted. No animals were used for this work. All studies were conducted on slaughterhouse-derived materials. The Gyeongsang National University Institute of Animal Care Committee approved all experiments including surgical procedures (GNU-130902-A0059).

### 2.1. Experimental Design

Cumulus–oocyte complexes (COCs) were collected from bovine ovaries and cultured in different IVM and IVC media compositions.

**First experiment**, COCs were cultured in IVM (control) and IVM + PHPS1 (5 μM SHP2 inhibitor). After maturation for 22 h, the oocytes were in vitro fertilized (IVF) in media for 18–20 h (untreated media in all groups and all experiments). The presumed zygotes were cultured in control IVC media and IVC + PHPS1 5 μM media for 8 days.

**Second experiment**, COCs were matured in control IVM and then IVC Control media was split into SOF (Synthetic ovarian fluid) and growth factors as:

1 SOF-BE1: SOF;
2 SOF + EGF + CIS: COMBO;
3 SOF + CIS: CISPLATIN;
4 SOF + EGF: CONTROL, as this is our standard control media.

**Third experiment**, EGF of the control media was replaced by fibroblast growth factor 2 (bFGF) (50 ng/mL) in IVM and SOF media according to previous studies [5]. IVM + bFGF and SOF + bFGF were considered as Control group and PHPS1 (5 μM) was added to both the media (IVM + bFGF + PHPS1 and SOF + bFGF + PHPS1) as experimental group.

**Fourth experiment**, the cytokine LIF was also treated to oocytes and zygotes as previously mentioned (25 ng/mL) [26]. In brief the IVM control group media composition was IVM + LIF (IVM Control) and treatment group IVM + LIF + PHPS1 (5 μM). The IVC control media composition was consisted of SOF + EGF + LIF and IVC treatment group media composition was consisted of SOF + EGF + LIF + PHPS1 (5 μM).

### 2.2. Oocyte Collection and In Vitro Maturation

Bovine ovaries were collected at a local abattoir as previously described [37]. The COCs were aspirated from 3 to 6 mm in diameter using an 18-gauge needle attached to a 10-mL disposable syringe. Collected COCs were allowed to settle down as sediment in 15 mL conical tubes at 37 °C for 5 min. TL-HEPES (114 mM sodium chloride (S-5886), 3.2 mM potassium chloride (P-5405), 2 mM sodium bicarbonate (S-5761), 0.34 mM sodium biphosphate (S-5011), 10 mM sodium lactate (L-4263), 0.5 mM magnesium chloride (M-2393), 2 mM calcium chloride (C-7902), 10 mM HEPES (H-6147), 1 μl/mL phenol red (P-0290), 100 IU/mL penicillin, and 0.1 mg/mL streptomycin) solution was used for the settled-down COCs, while the supernatant was discarded. After that, under a stereomicroscope, the COCs were recovered. Only COCs having ≥3 uniform layers of compact cumulus cells were selected and washed three times with TLH-PVA (P-8136). Approximately 50 COCs were placed into each well of a four-well Nunc dish (Nunc, Roskilde, Denmark) containing 700 μL of IVM medium (TCM199; Invitrogen Corp., Carlsbad, CA, USA) with 10% (*v*/*v*) FBS (Fetal bovine serum) Gibco BRL, Life Technologies, Grand Island, NY, USA cat.# 16000-044), 1 μg/ml oestradiol-17β, 10 μg/mL follicle-stimulating hormone (ProSpec USA cat.#HOR-285), 0.6 mM cysteine, 10 ng/mL epidermal growth factor, and 0.2 mM sodium pyruvate (Gibco BRL, Life Technologies, Grand Island, NY, USA cat.#11360-070) for 22–24 h at 38.5 °C in a humidified atmosphere of 5% CO_2_ in air.

### 2.3. In Vitro Fertilization and In Vitro Culture

IVM COCs were fertilized with frozen–thawed bovine sperm, as previously described [38]. In brief, semen was thawed at 39.0 °C for 1 min and the sperm were washed in D-PBS, followed by centrifugation at 750 × *g* for 5 min at room temperature. The supernatant was discarded and the pellet was diluted with 500 μL of heparin (20 μg/mL) in IVF media (Tyrode lactate solution supplemented with 6 mg/mL BSA, 22 μg/mL sodium pyruvate, 100 IU/M penicillin, and 0.1 mg/mL streptomycin) incubated at 38.5 °C in a humidified atmosphere of 5% CO_2_ air for 15 min to facilitate capacitation. Thereafter, sperm were diluted in IVF medium (final density of 1–2 × 10^6^ sperms/mL). After coculture with spermatozoa for 20 h, cumulus cells were removed by pipetting and groups of up to 50 presumed zygotes were washed and transferred to four-well dishes containing 500 mL of SOF-BE1 medium supplemented with 4 mg/mL fatty-acid-free BSA, 5 μg/mL insulin, 5 μg/mL transferrin, and 5 ng/mL sodium selenite and cultured until day 8 of embryonic development.

### 2.4. Histological Analysis

Bovine ovaries were collected in saline followed by washing with 1 × PBS and put in 20% sucrose in 1 × PBS solution for 72 h, and after that, for 72 h in 4% paraformaldehyde. After that, optimal cutting temperature (OCT) compound (Sakura Finetek Inc., Torrance, CA, USA) was used for freezing and blocking the ovaries at −80 °C. From each block, 12-μm sections were cut using a CM 3050C cryostat (Leica, Germany). The ovary sections were taken on probe-on plus charged slides (Fisher, Rock-ford, IL, USA). The slides were stored at −80 °C until further processed.

### 2.5. Immunofluorescence

Immunofluorescence staining was performed as previously discussed [38,39]. Briefly, oocytes or blastocysts were fixed in 4% (*v*/*v*) paraformaldehyde prepared in 1 M phosphate-buffered saline (PBS) and preserved at 4 °C. On staining day, oocytes or blastocysts were taken in four-well dishes and washed twice in 1 × PBS for 10 min. Proteinase K solution was then added for 5 min to retrieve the antigen. Subsequently, the blastocysts were incubated for 30 min in blocking solution containing normal bovine or donkey serum and 0.1% Triton X-100 in PBS. Primary antibodies were applied and the four-well dishes were kept at 4 °C overnight. Next day, the blastocysts or oocytes were washed twice with 1 × PBS for 10 min. After washing, secondary antibodies (FITC and TRITC conjugated, Santa Cruz Biotechnology, USA) were applied at room temperature for an additional 90 min. Blastocysts and oocytes were washed again three times with PBS for 5 min. After that, blastocysts and oocytes were treated with 4′, 6′-diamidino-2-phenylindole (DAPI) for 10 min to stain the nucleus and fixed on slides. The slides containing ovarian tissues were washed twice with 1X PBS for 10 min and Proteinase K solution was used for 5 min to permeabilized ovarian tissue. After that blocking with donkey serum and 0.1% Triton X-100 in 1 × PBS. Primary, secondary antibodies and DAPI were used as mentioned above with same washing durations. Thereafter the slide containing blastocysts, oocytes, and ovary tissues were covered with glass coverslips using fluorescent mounting medium. Images were captured with a confocal laser-scanning microscope (Fluoview FV 1000, Olympus, Tokyo, Japan). To measure the relative integrated density, the signal and area were obtained by using the ImageJ analysis program (version 1.50, National Institutes of Health, Bethesda, MD, USA; https://imagej.nih.gov/ij).

### 2.6. TUNEL Assay

The TUNEL assay was performed according to the manufacturer's protocols using an In Situ Cell Death Detection Kit (Roche Diagnostics Corp., Indianapolis, IN, USA). Briefly, fixed embryos were washed twice with 0.3% (*w*/*v*) polyvinylpyrrolidone (PVP) prepared in 1 × PBS (PVP-PBS) before being permeabilized (0.5% (*v*/*v*) Triton X-100 and 0.1% (*w*/*v*) sodium citrate) at room temperature for 30 min [40]. After that, the blastocysts were incubated in the dark with fluorescent conjugated terminal deoxynucleotidyl transferase dUTP at 37 °C for 1 h. Stained embryos were then washed with PVP-PBS and incubated in Hoechst 33342 (10 μg/mL) for 10 min. After washing with PVP-PBS, blastocysts were mounted onto a glass slide and their nuclear configuration was analyzed. The number of cells per blastocyst was determined by counting Hoechst-stained cells under an epifluorescence microscope (Olympus IX71, Tokyo, Japan) equipped with a mercury lamp. The TUNEL-positive cells were labeled bright red (apoptotic) and normal cells were labeled blue.

### 2.7. H2DCFDA Assay (Reactive Oxygen Species (ROS) Detection)

ROS was measured through 2,7,dichlorodihydrofluorescein diacetate (H_2_DCFDA) as described previously [38]. In brief, the live mature (MII) oocytes (30 to 35 used per each group/experiment) or day-8 blastocysts (10 to 15 used per each group/experiment) were incubated in 1 × PBS containing 10 μM of H_2_DCFDA for 30 min in a humidified atmosphere of 5% (*v*/*v*) CO_2_ in air at 38.5 °C. When the incubation time was finished, the COCs were washed three times with PBS, mounted onto glass slides, and examined under an epifluorescence microscope (Olympus IX71, Tokyo, Japan) under 490 nm excitation and 525 emission.

### 2.8. Extraction of mRNA and cDNA Synthesis

Total mRNA was extracted as previously described [41]. In brief, mRNA was extracted at different biological replicates, with 5 blastocysts or 20 oocytes per replicate (day 8, n = 16 per group), using a Dynabeads mRNA direct kit (Dynal AS, Oslo, Norway). In 100 μL of lysis buffer, oocytes or blastocysts were suspended and vortexed at room temperature for 2 min. The lysate was mixed with prewashed Dynabeads oligo (dT) (20 μL) and annealed by rotation at room temperature for 3 min. The Dynal MPC magnetic particle concentrator was used to remove the supernatant. The magnetic beads harboring the hybridized mRNA and oligo (dT) were washed twice with 300 μL of washing buffer A and twice with 150 μL of washing buffer B. To denature the secondary structures, bound mRNAs were resuspended in 8 μL of 10 mM Tris-HCl and heated at 65 °C for 5 min, followed by rapid quenching of the reaction on ice for 3 min. Superscript III reverse transcriptase was used for mRNA to reverse-transcribe into the first-strand cDNA. The final reaction volume was increased to 80 μL by adding RNase-free water. The primers and PCR conditions for each gene are given below (Table 1).

### 2.9. Real-Time Polymerase Chain Reaction

As previously described [41,42], complementary DNA samples were subjected to RT-PCR using glyceraldehyde-3-phosphate dehydrogenase (GAPDH) primers to test for any variation in the expression of this internal control gene. After confirming that there was no significant difference in the relative expression of GAPDH among samples, all transcripts were quantified using independent qRT-PCR reactions. The cycles were as follow: At 95.0 °C for 3 min, followed by 44 cycles at 95.0 °C for 15 s and at 72.0 °C for 30 s, and a final extension at 72.0 °C for 5 min. Amplification was followed by melting curve analysis using progressive curve analysis. Using progressive denaturation, the temperature was raised from 65 to 95 °C at a transition rate of 0.2 °C/s. Continuous fluorescence QuantiTect^®^SYBR Green PCR Master Mix measurements were acquired during incremental heating. Final quantitative analysis was performed by the ^ΔΔ^CT method, and results are reported as the relative expression to the calibrator after normalization of the transcript to the average value of the endogenous control, GAPDH. The coefficients of variation (CV) of the intra- and interassay variance were calculated according to the formula SD/mean × 100 for all genes profiled with RT-PCR.

### 2.10. Invasion Assay

For invasion quantification, day-8 blastocysts were cultured on Matrigel invasion chamber inserts (6.4 mm; Corning Inc. Life Sciences USA) containing polyethylene terephthalate membranes with 8-mm-diameter pores in 24-well tissue culture plates (Corning Inc. Life Sciences Corning, New York, NY, USA) [43]. The filters were coated with Matrigel (20 mg per filter; Discovery Labware Inc. Billerica, MA, USA) and then incubated at 37.8 °C for 2 h to dry. The day-8 blastocysts were cultured on the filter coated with Matrigel (three blastocysts per culture insert suspended in the same medium used for embryo production), then incubated under a humidified atmosphere of 5% CO_2_ in air at 37.0 °C for 72 h. After that, the culture medium at the bottom of the culture was changed from IVC1 to IVC2 and refreshed on a daily basis [44]. At day 10 of culture, the invasion area of the trophoblasts was evaluated under a phase contrast Olympus IX71 microscope and measured using ImageJ software. Thereafter, the upper surface of the chamber insert membrane was scrubbed three times with a cotton swab. The cells on the lower surface of the scrubbed membrane were fixed with 4% (*v*/*v*) paraformaldehyde prepared in 1 M PBS for 15 min at room temperature and then stained with DAPI for 5 min. Cells that traversed the membrane were counted under a phase-contrast Olympus IX71 microscope at 100× total magnification.

### 2.11. Protein Extraction

Proteins were extracted from bovine ovaries (three per extract) as previously described [45] with some minor modifications. Briefly, total proteins were extracted from ovaries using protein extraction solution pro-prep™ (iNtRON Biotechnology, Burlington, NJ, USA cat. #17081) according to the provided instructions. The samples were homogenized with homogenizer (iNtRON, Biotech, Inc. Seoul, South Korea) and cell lysis was induced by incubating the cells on ice for 30 min. The lysate (protein homogenate) solutions were centrifuged at 13,200 rpm for 25 min at a controlled temperature (4 °C). The protein (supernatants) were collected and placed at −80 °C for further analysis. Similarly, day-8 blastocysts (20 blastocysts per extract) were washed with PBS, dissolved thoroughly in pro-prep™ sonicated to make cell lysates, and then centrifuged at 13,200 rpm at 4 °C for 25 min. The supernatant was stored at −80 °C for further analysis.

### 2.12. Western Blot Analysis

The protein extract of lysed ovaries (three per extract) and blastocysts (20 per extract) concentrations were quantified with a Bradford assay as previously described [45,46], with some minor modifications. Briefly, Bio-Rad protein assay kit (Bio-Rad Laboratories, Hercules, CA, USA cat. # 5000002) was used to measure the concentration of proteins in the homogenates. Equal amounts of proteins (25 μg) were fractioned by 12% SDS Polyacrylamide gel, transferred to a Polyvinylidene difluoride membranes (PVDF) (sigma-Aldrich, St. Louis, MO, USA cat. # GE10600023) membrane, and blocked in 5% skim milk or 5% BSA (1×TBST (1×Tris-Buffered Saline, 0.1% Tween 20 Detergent)) before incubation with primary antibodies overnight at 4 °C. On day two the PVDF membrane was washed with 1×TBST to remove unbounded primary antibody and incubated with secondary antibody for 1 h at room temperature. Next after washing thrice with 1X TBST the proteins were detected using an ECL (Pierce ^TM^ ECL Western Blotting Substrate ThermoFisher Scientific) detection reagent according to the manufacturer’s instructions. Prestained protein ladders (abcam, USA cat. # ab116029) covering a broad range of molecular weights were used to detect the molecular weights of the proteins. The X-ray films (iNtRON, Biotechnology Inc.) were scanned, and the optical densities of the bands were analyzed via densitometry using the computer-based ImageJ program (National Institutes of Health, Bethesda, MD, USA; https://imagej.nih.gov/ij).

### 2.13. Antibodies

The following antibodies from SANTA CRUZ biotechnology (USA) were used in this study: rabbit-derived anti-mouse p-PI3K (cat. # sc-374534), anti-mouse COX2 (cat. #sc-7951), anti-mouse SH-PTP2 (cat. #sc-271106), anti-mouse Caspasae3 (cat. #sc-1225), anti-mouse p-NF-κB (cat. #sc-271908), anti-mouse OCT4 (cat. #sc-8629), mouse β-actin (cat. # sc-47778), while anti-rabbit SHP2-Y580 (Mybiosource cat. #MBS9601182), anti-mouse p-AKT (cell signaling cat. #9271), anti-rabbit H3K56ac (abcam, USA cat. #ab71956), Anti-Steroidogenic Factor 1 (abcam, USA cat. #ab168380) and p-STAT3 Y705 (Cell Signaling cat. #9131S).

### 2.14. Statistical Analysis

A computer-based Sigma Gel System (SPSS software Inc., Chicago, IL, USA) was used for embryo development analysis. GraphPad Prism 6 (GraphPad Software, San Diego, CA, USA.) and the Image J program (USA) were used to analyze the density and integral optical density (IOD) of scanned X-ray films of Western blot and immunofluorescence images. One-way ANOVA followed by Student’s t-test was used to determine the statistical significance (*p*-value) of the obtained data. The density values of the data are expressed as the mean ± SEM of three independent experiments. Significance: * = *P* < 0.05, ** = *P* < 0.01, and *** = *P* < 0.001.

## 3. Results

### 3.1. SHP2 Expression in Bovine Ovary, Oocytes and Blastocysts

**Figure 1:**
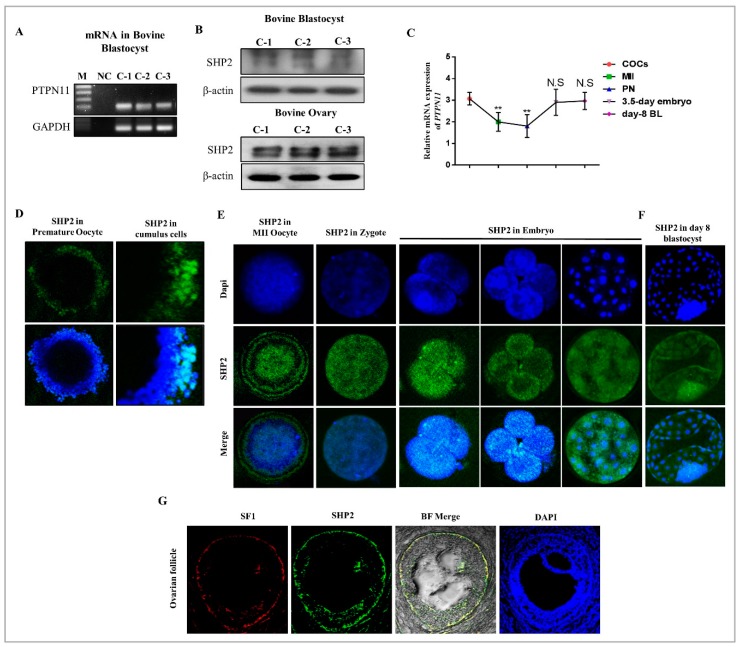
The qualitative expression of SHP2 in bovine oocytes, blastocysts and ovarian tissues. (**A**) PTPN11 mRNA expression was qualitatively analyzed in bovine day 8 blastocysts from three independent control groups with 100 bp (n = 5 per each group). GADPH was used as a housekeeping gene. (**B**) SHP2 protein expression was assessed via western blot from three independent control groups (C1, C2 and C3) in bovine day 8 blastocysts (n = 20) and ovaries (n = 3) at 90 kDa. β-actin was used as a loading control for western blot, and the experiments were repeated three times. (**C**) Quantitative real time RT-PCR analysis was performed for PTPN11 in GV oocyte (n = 20), MII oocyte (n = 20), Pronuclear zygote (PN) (n = 20), day 3.5 embryo (n = 5) and day 8 blastocyst (n = 5). (**D**) Representative image of immune labeling of SHP2 (green) in GV oocyte and cumulus cells from COCs (n = 20). (**E**) Immunoreactivity of SHP2 (green) in MII oocyte (n = 20), Zygote (n = 20), 2 cells (n = 20), 4 cells (n = 20) and day 3.5 embryo (n = 20). (**F**) SHP2 expression in bovine day 8 bovine blastocyst (n = 20). (**G**) Immunoflourscent co-localization of SF-1 (red) and SHP2 (green) in bovine pre-ovulatory follicle (n = 5). Image J was used to quantify the signal intensity of immunofluorescence images. All the experiments were repeated 3 times and the data are shown here as a mean ± S.E.M. * *p* < 0.05, ** *p* < 0.01, *** *p* < 0.001.

As a first step toward understanding the role of SHP2, we qualitatively assessed the mRNA expression of PTPN11 (encoding protein SHP2) in bovine day-8 blastocysts (Figure 1A). After that SHP2 protein expression was qualitatively analyzed through western blot in bovine day-8 blastocysts and ovaries (Figure 1B). To define the cellular events in which SHP2 is involved during meiotic maturation and embryo development, we examined the distribution of SHP2 at different developmental stages through qRT-PCR and immunofluorescence (Figure 1C,D). The results indicate that SHP2 was undetectable at GV stage, but the immunolabeling identified the expression in surrounding cumulus cells of GV oocyte (Figure 1D, Figure S1A,B). Accompanying the meiotic resumption, SHP2 resides in the entire oocyte and become enhanced as the developmental stages proceeded from zygote to the day-3.5 stage embryo (Figure 1E). Interestingly SHP2 show high expression in the ICM as compare to other cells of day-8 blastocyst (Figure 1F). To investigate SHP2 expression in theca cells, the pre-ovulatory phase of oocyte was targeted in bovine ovary. In order to get theca cells specific SHP2 expression, immunofluorescent staining was done for SHP2 and SF1 (Nr5a1) (a main marker for theca cells), and the result indicate the expression of SHP2 in theca cells (Figure 1G) [47]. This data illustrated that SHP2 is expressed in thecal cells of pre-ovulatory follicle and cumulus cells of COCs, but undetectable in GV oocytes. PTPN11 (SHP2) gene start its expression during maturation and elevates with the development. Furthermore, SHP2 become expressed in all cells of bovine day 8 blastocyst, but the expression was apparently high in ICM.

### 3.2. SHP2 Inhibition Compromise Oocyte Maturation and Embryo Development

**Figure 2:**
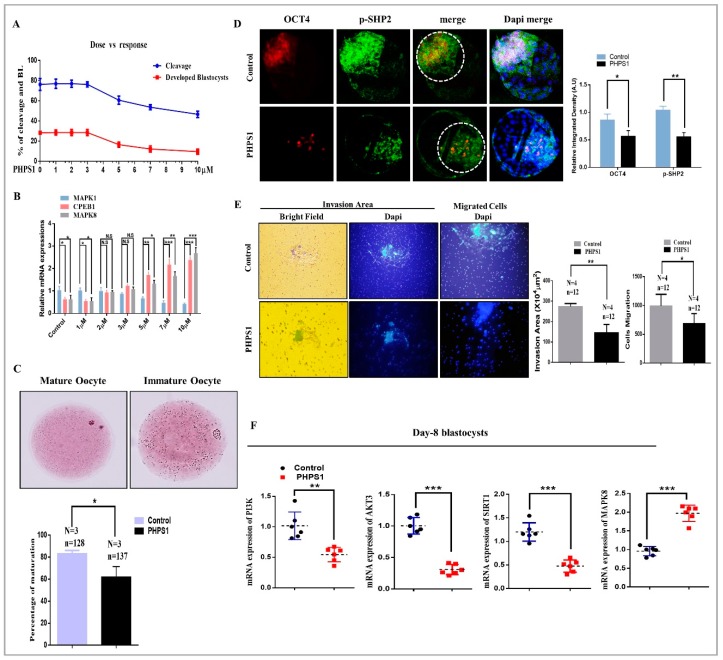
The effects of SHP2 inhibition was analyzed during oocytes maturation and blastocysts development. (**A**) Dose dependent response of PHPS1 (SHP2 inhibitor) toward embryo cleavage and blastocysts development. (**B**) Dose dependent effect of PHPS1 on oocyte maturation pathways. Increase in PHPS1 concentration reduced mRNA expression of MAPK1, while CPEB-1 and MAPK8 showed upregulated expression (n = 20 per each group). (**C**) Aceto-orcein staining for detection of germinal vesical breakdown (GVBD) and first polar body (PB1) extrusion (oocyte maturation). Independent experimental repeat (N) and used oocytes (n) values are as indicated. SHP2 inhibition prevent oocyte maturation, indicating 62% maturation in PHPS1 group as compare to control vehicle-treated 83% maturation. Data are expressed as means ± SEM. Control and SHP2 inhibited blastocysts were immunolabeled with OCT4 (red) and SHP2 (green) and counter stained with DAPI to visualize DNA (Mean ± SEM). (**D**) Immunolocalization showing reduced OCT4 (red) and SHP2 (green) expression in PHPS1 treated blastocysts as compare to vehicle-treated control (n = 20 per each group). (**E**) The effects of SHP2 inhibition was checked for blastocysts implantation potential. Bright field showing area of invasion and DAPI for migrant cells in day-8 blastocysts. The invaded area and cellular migration were significantly lower in PHPS1 treated group as compare to control vehicle-treated group. Image J software was used to quantify the signal intensity of immunofluorescence images. (**F**) Genes related to survival and apoptosis were examined through qRT-PCR in day 8 blastocyst (n = 5). SHP2 inhibition significantly reduced the PI3K, AKT and SIRT expression, while substantially enhanced the apoptosis signaling related gene MAPK8 (JNK). The experiments were repeated 3 times and the data are shown here as a mean ± S.E.M. * *p* < 0.05, ** *p* < 0.01, *** *p* < 0.001.

The specific subcellular localization of SHP2 led us to speculate that SHP2 has some special role in bovine oocyte maturation and embryo development. So, we used various concentrations of PHPS1 and checked percent of embryo cleavage and developed blastocysts percentage. The results showed the reduction in cleavage and development percentage with the increase in PHPS1 concentration (Figure 2A). To confirm our effective concentrations, we treated GV oocytes again with same concentrations of PHPS1 and quantified the mRNA expression of MAP kinases and CPEB-1 (Figure 2B). SHP2 inhibition abrogated MAPK1 signaling as a result high CPEB-1 expression reduce oocyte maturation as compared to control, where the activated SHP2 enhanced MAPK1 activity and reduce CPEB1 (Figure 2B) [6,7]. Inhibition of SHP2 also enhanced MAPK8 a member of second MAP kinase family (JNK1, 2 and 3), which indicate the effect of SHP2 inhibition on apoptosis. From above results, we selected 5 μM as minimum effective concentration. To confirm SHP2 inhibition reduce oocyte maturation we used aceto-orcein staining. The oocytes with extruded first polar body was 62% in PHPS1 group as compare to control 83% (Figure 2C). Next to determine whether apoptosis in SHP2 inhibited oocytes were dependent on ROS level, we stained oocyte with H2DCFDA and elevated ROS level was observed in PHPS1 group (Figure S2A). Excessive ROS level also reduce fertilization rate, and this phenomenon was observed in PHPS1 group (Figure S3A). The polyspermic oocytes number was non-significant in both groups (Figure S3B). Given the effects of SHP2 inhibition on oocyte quality, we asked whether inhibition of SHP2 would thereby impair the developmental competence of subsequent zygotes. To do this we added PHPS1 to IVC media and cultured zygotes for 8 days. Inhibition of SHP2 reduced embryo cleavage to 50.00% as well as blastocysts development to 16.14%, as compare to control cleavage 75.71% and control blastocysts development percentage 31.86% (Table 2).

The size of blastocyst as well as the number of cells per blastocyst, were substantially reduced with SHP2 inhibition (Figure S3B). Furthermore, we examined blastocyst ICM volume by co-expressing SHP2 with OCT4 through immunofluorescence and the result showed marked reduction in ICM volume with PHPS1 treatment (Figure 2C). SHP2 has well known role in cellular invasion, migration and proliferation, so this activity was checked for blastocyst implantation by using invasion assay [48]. The invasive area and proliferation activity in the control verses PHPS1 groups were quantified, which indicated that SHP2 inhibition reduce blastocyst potential of maternal tissue invasion (Figure 2D) [49]. It was speculated that the reduction in development percentage might be due to the reduced expression of RTK (Receptor Tyrosine Kinase) downstream proteins at embryo stage. To address this question the mRNA expression of intracellular RTKs receptor signaling genes were examined through qRT-PCR in day 8 blastocysts. As shown in Figure 2E, the mRNA expression of PI3K, AKT, and SIRT-1 were significantly upregulated in control group as compare to PHPS1 treated group, where the mRNA expression of MAPK8 was markedly enhanced in PHPS1 treated group. On the basis of these findings, we can say that SHP2 depletion reduce RTK receptor downstream proteins and enhance apoptosis during oocyte maturation. Moreover, in bovine embryo SHP2 inhibition has the same lethal effects as previously observed in SHP2 knockout mouse embryos [50].

### 3.3. EGF Neutralize Cisplatin Induced Apoptosis and Enhance Rate of Blastocysts Development

**Figure 3:**
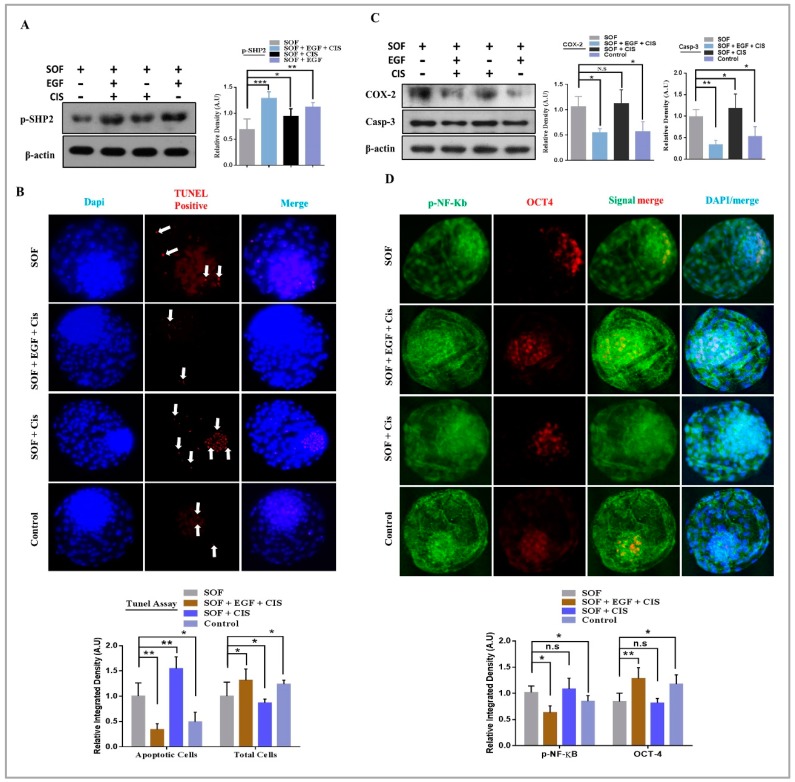
EGF neutralize cisplatin induced apoptosis and enhances the developmental rate of blastocysts. (**A**) Western blot analysis of SHP2 in blastocysts grown in SOF, COMBO, CISPLATIN and Vehicle-treated CONTROL media (n = 20 per each group). β-actin was used as a loading control for western blot. The bands were quantified using ImageJ software, and the differences are represented by histogram. (**B**) TUNEL assay was performed to detect apoptotic cells. TUNEL positive cells were markedly enhanced in CISPLATIN group and were mostly present in the ICM as compare to SOF, COMBO and vehicle-treated CONTROL groups (n = 20 per each group). (**C**) Western blot of COX-2 and Caspase-3 protein expression showing high apoptosis in CISPLATIN group as compare to other groups (n = 20 per each group). β-actin was used as a loading control for western blot. The bands were quantified using ImageJ software, and the differences are represented by histograms. (**D**) Blastocysts were costained with OCT4 (red) and p-NF-κB (green) for immunofluorescence to analyze ICM and apoptosis. OCT4 retain its level while nuclear localized p-NF-κB was non-significantly in the COMBO as compare to CONTROL group. The experiments were repeated 3 times and the data are shown here as a mean ± S.E.M. N.S, not significant. * *p* < 0.05, ** *p* < 0.01, *** *p* < 0.001.

To delineate the mechanism of SHP2 with growth factors during embryo development, the IVC control media was split into basic media (SOF) and growth factors. SHP2 selective activator Cisplatin (Figure S2C, 1.4 μM) was added to the media and presumed zygotes were developed in media with various factors combination. For simplicity, the different media combinations were named as IVC media without growth factors was SOF group, while SOF + CIS group was named cisplatin group, SOF + EGF + CIS was name combo group, and the last one was control group SOF + EGF (our standard IVC media). In SOF group the developed blastocysts were 20.25% and surprisingly reduction in development rate was observe in cisplatin group 18.00% (Table 3). In combo group the blastocysts development was highly enhanced 41.00% as compare to control group 31.25%. Protein expression of p-SHP2 was analyzed through western blot in all of the above media compositions developed blastocysts. High p-SHP2 expression in combo group compelled us to accredited enhanced rate of blastocysts development to SHP2 (Figure 3A). In cisplatin group, the blastocysts development percentage was reduced but the expression of SHP2 shown significant elevation compared to SOF group.

To identify the mechanism behind this phenomenon, a TUNEL assay was performed. As shown in Figure 3B, blastocyst with high amount of apoptotic positive cells in cisplatin group and the apoptotic signal were mostly reside in the ICM. Similarly, western blot expression of COX2 and caspase 3 also suggested that cisplatin enhanced apoptotic proteins expression in the absence of EGF (Figure 3C). However, in combo group, this apoptotic effect was effectively neutralized, as previously reported that EGF neutralizes the interferon apoptotic effect via RAS and ERK1/2 pathways [51,52]. Earlier it has been investigated that cisplatin have ICM damaging effect and our TUNEL assay result also showed enhance apoptosis in ICM [53]. So here the question arise what would be the effect of cisplatin in the presence of EGF? To address this question the blastocysts were stained with OCT4 and p-NF-κB antibodies to analyze the cisplatin ICM damaging effect in the presence and absence of EGF. There was a significantly low OCT4 expression in cisplatin group with high nuclear localized p-NF-κB in cisplatin group as compare to other groups (Figure 3D). So, cisplatin enhance apoptosis and damage ICM in absence of growth factors, but presence of EGF in IVC media significantly neutralize the apoptotic and ICM damaging effects of cisplatin. Combine treatment efficiently transduce EGF receptor signaling and result in enhanced rate of blastocysts development.

### 3.4. Consistent SHP2 Expression in the Presence of Ligand, Enhance RTK Downstream Pathway Proteins Without Effecting Chromatin Stability

**Figure 4:**
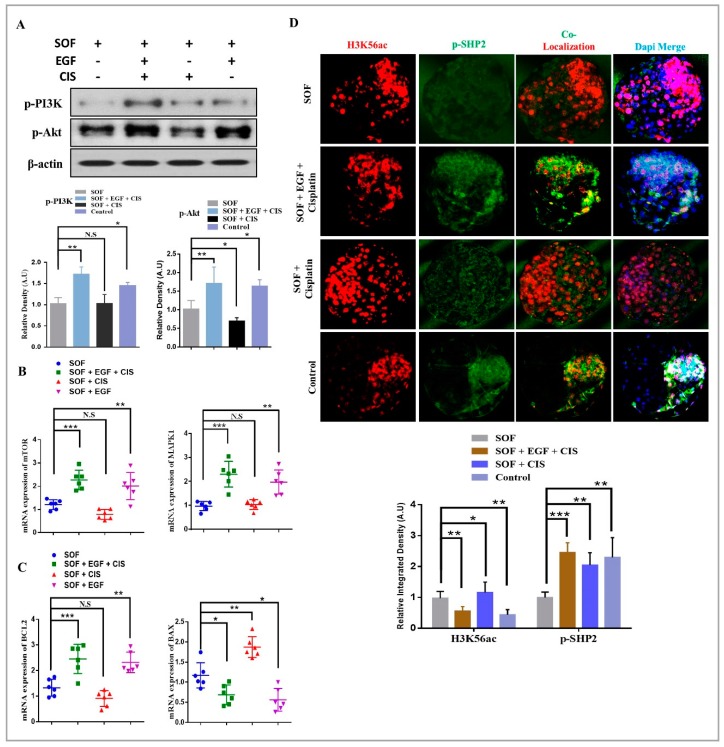
Consistent SHP2 expression enhance RTKs downstream signaling and chromatin stability. (**A**) Western blot of PI3K and p-AKT protein expression level was significantly enhanced in the presence of EGF in both COMBO and CONTROL groups. β-actin was used as a loading control for western blot. The bands were quantified using ImageJ software, and the differences are represented by histograms. (**B**) Relative mRNA expressions of mTOR and MAPK1 in blastocysts (n = 5 per each group). (**C**) Relative mRNA expressions of BAX and BCL2 in blastocysts (n = 5 per each group). (**D**) Immunofluorescent co-localization of H3K56ac with SHP2 in bovine blastocysts (n = 20 per each group). The experiments were repeated 3 times and the data are shown here as a mean ± S.E.M. N.S, not significant. * *p* < 0.05, ** *p* < 0.01, *** *p* < 0.001.

We then sought to examine the effect of cisplatin on EGFR intracellular downstream signaling in the presence and absence of ligand. EGFR mainly activate PI3K/AKT and MAPK3/1 signaling pathways [54]. So, we quantified immunoblot expression of p-PI3K and p-AKT (ser 473 and thr 308) in all the media combination groups. As shown in Figure 4A, the protein expression of p-PI3K and p-AKT were markedly enhanced in combo group as compare to SOF, cisplatin and control groups. Next, we assessed the mRNA expressions of MAPK1 and mTOR and both the genes showed marked enhancement in combo group as compare to control, SOF and cisplatin groups (Figure 4B). After that we examined the effect of cisplatin on mitochondrial activity of blastocysts. To do this we quantified mRNAs of BCL-2 family through qRT-PCR. The abundant BCL-2 mRNA with low BAX expression in combo and control groups was found, whereas in cisplatin group the elevated expression of BAX as compare to SOF and other groups indicate some mitochondrial damage occurred by cisplatin in the absence of EGF (Figure 4C). Cisplatin DNA-damaging effect and genomic instability was previously reported [55], but again the question arise whether presence of growth factor can modulate cisplatin mode of action? So, blastocysts were stained with H3K56ac along with SHP2 antibody for immunofluorescence [56,57]. As shown in Figure 4D, cisplatin group showed an enhanced acetylated H3K56 expression as compare to combo, SOF and control groups. The expression of SHP2 was high in cisplatin group as compare to SOF group, but the expression was not ICM localized like in combo and control blastocysts. Thus, the expression of p-PI3K, p-AKT, mTOR and MAPK1 suggest that cisplatin activated SHP2 is not sufficient, but it acts as a mediator of EGFR signaling in the presence of ligand, so that these signals could attain sufficient levels to induce the corresponding cellular responses and improve rate of development.

### 3.5. SHP2 is Essential for RTK and Cytokine Receptor Signal Transduction During Early Developmental Stages 

**Figure 5:**
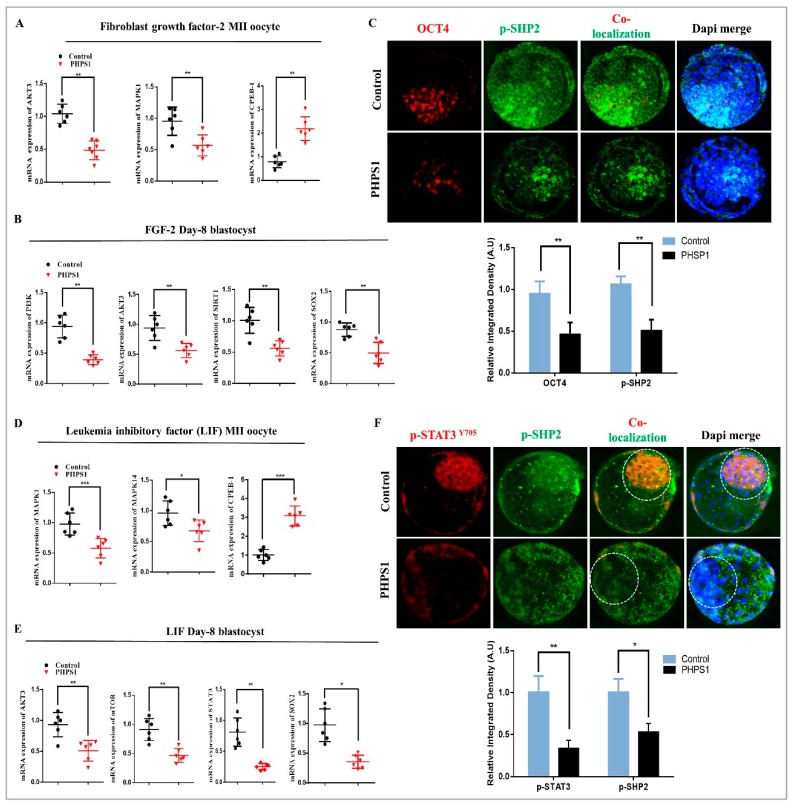
SHP2 dependent RTKs and Cytokines signal transduction. (**A**) FGF2 and PHPS1 treated groups showing relative mRNA expressions of AKT3, MAPK1 and CPEB1 in oocytes (n = 20 per each group). (**B**) FGF2 and PHPS1 treated groups showing relative mRNA expressions of PI3K, AKT3, Sirt1 and SOX2 in blastocysts (n = 5 per each group). (**C**) Immunofluorescent co-localization of SHP2 and OCT4 in blastocysts (n = 5 per each group) treated with FGF2 and PHPS1. (**D**) Relative mRNA expression level of MAPK1, MAPK14 and CPEB1 in oocytes treated with LIF and PHPS1 (n = 20 per each group). (**E**) Relative mRNA expression level of AKT3, mTOR, STAT3 and SOX2 in blastocysts treated with LIF and PHPS1 (n = 5 per each group). (**F**) Immunofluorescent co-staining of p-STAT3 and p-SHP2 protein expression in blastocysts (n = 20 per each group). Image J software was used to quantify the signal intensity of immunofluorescence images. The experiments were repeated 3 times and the data are shown here as a mean ± S.E.M. N.S, not significant. * *p* < 0.05, ** *p* < 0.01, *** *p* < 0.001.

Evidence indicates that SHP2 is not only restricted to EGF, but it also plays an essential role in signal transduction of other growth factors and also cytokines [24,26]. So, we selected two most frequently used factors FGF2 and LIF and investigated their dependency on SHP2 for intracellular signaling during oocyte maturation and embryo development [26,58]. First, we examined the effect of FGF2 by replacing it with EGF in maturation media and IVC media, as previously studied [58]. The oocytes and zygotes were grown in both media with or without PHPS1, and the percentage of blastocysts development indicate that SHP2 inhibition block FGF2 dependent embryos development (Table 4). Previous study indicated that FGF2 enhance MAP kinases [59], so we assessed the mRNA levels of MAP kinases AKT3 and CPEB-1 for oocyte maturation. As shown in Figure 5A, SHP2 inhibition during maturation decrease the level of MAPK1 and AKT3 mRNAs, whereas the CPEB-1 was significantly increased. Next, SHP2 inhibition effect in the presence of FGF2 was analyzed in blastocysts. FGF2 is RTK member so again PI3K/AKT and one another target of this pathway SIRT1 was analyzed. Remarkably, the pathway genes and SIRT-1 were significantly decreased with SHP2 inhibition (Figure 5B). Furthermore, we examined PHPS1 effect on ICM related genes SOX2 and OCT4 in the presence of FGF2. OCT4 was co-localized with SHP2 and the result showed reduced and dispersed expression of OCT4 in PHPS1 group (Figure 5C).

We next examined SHP2 dependent LIF intracellular signaling in bovine oocytes and embryos. Early investigations suggested that LIF activate two signaling cascades in bovine oocyte and embryo MAPK3/1 and JAK/STAT3 [26]. So first we analyzed cleavage and development and the result indicated low cleavage (57.89%) and developed blastocyst (13.67%) as compare to control LIF cleavage (81.78%) and developed blastocyst (33.33%) (Table 4). Next, we moved toward MAP Kinases quantification through qRT-PCR and we found significant dropdown in the mRNAs of MAPK1 and MAPK14 and enhancement in CPEB-1 mRNA in PHPS1 group (Figure 5D). Similarly, SHP2 inhibition reduced blastocysts AKT, mTOR and SIRT-1 mRNAs and ICM related genes STAT3, SOX2 and OCT4 (Figure 5E, Figure S2C) [60]. SHP2 knockout reduce embryo development and STAT3 is critical for bovine ICM development [50,61]. Normally SHP2 inhibit p-STAT3 nuclear translocation by dephosphorylation it [14,62]. To find out behavior of both proteins in bovine blastocysts, we costained day-8 blastocysts with p-STAT3 and p-SHP2 for immunofluorescence and found a surprising relation. As shown in Figure 5F, inhibition of SHP2 highly decreased and scattered the p-STAT3 expression as compare to control where both proteins reside in the ICM. Overall, our findings suggested that receptor tyrosine kinases (EGFR and FGFR) and cytokine like LIF depend on SHP2 for their signal transduction during oocyte maturation and blastocyst development.

## 4. Discussion

In the present study, we systemically explored the expression characteristics and function of SHP2 in bovine oocytes and embryos. We showed that growth factors and cytokines activate SHP2 for their downstream signaling, which is not only important for oocyte maturation, but also for proper embryonic development. Inhibition/activation of SHP2 highly influence invitro oocyte maturation as well as embryonic development.

SHP2 is a nonreceptor phosphatase containing two Src homology domains (SH2), one PTP domain, and a proline-rich sequence along with tyrosine phosphorylation sites in its extreme C-terminal region [63]. SH2 domains and phosphatase are essential for SHP2 biological functions, as SH2 domain homologous deleted embryos have been shown to die in the uterus at embryonic day 10.5 from multiple defects [64,65]. Our finding that SHP2 play an essential role in early embryo development is consistent with previous work done in mouse embryos in which SHP2 deletion leads to ICM death, reduced trophoblast giant cells, and failure to yield trophoblast stem cells (Figure 2D and Figure 5C) [50]. SHP2 showed its expression in bovine ovaries, and theca cells of pre-ovulatory follicles, was not surprising (Figure 1) [66]. As it has been identified that *PTPN11* mRNA become significantly enhanced by inhibiting Poly (ADP-ribosyl) ation in the mouse ovary, which leads to enhanced number of primordial follicles and ovulated oocytes [66]. The interesting thing, we found was SHP2 nuclear localization in theca cells of pre-ovulatory follicles and in cumulus cells of COCs (Figure 1D,G). SHP2 nuclear localization in mice uterus play an important role in embryo implantation, by enhancing progesterone receptor [67]. Progesterone receptor inhibition in COCs reduced oocyte maturation [68] and SHP2 inhibition also reduced oocyte maturation (Figure 2C), which provide evidence of SHP2 importance for oocyte maturation. However, nuclear localized SHP2 in theca and cumulus cells needs exploration. 

In vitro oocyte maturation is a complex phenomenon and legend activated RTKs and cytokine receptors highly influence inter and intracellular signaling of COCs [69]. RTKs inhibitor significantly reduced oocyte maturation and embryo development [70] and SHP2 catalytic activity is essential for the regulation of downstream signaling of RTKs and cytokine receptors (Figure 4) [16,17]. Also, SHP2 knockout embryos showed deregulated tyrosine kinase signaling during development [65]. Liang, C.G. et., stated that oocyte meiotic resumption need balance between kinases and phosphatases and SHP2 expression in cumulus cells and MII oocyte support that statement and also its significance for GVBD induction (Figure 1D,E) [71]. EGF receptor activation highly influence bovine oocyte maturation by activating MAP kinases and SHP2 phosphatase activity inhibition markedly reduced MAP kinases during oocyte maturation and also during embryo development (Figure 2C,D) [8,9]. FGF receptors, other members of RTK superfamily also play an essential role in the embryo development and inner cell mass segregation [72]. SHP2 is essential for FGF signal transduction, as SHP2 dephosphorylate and inhibit Spry, which is the conserved inhibitor of FGF receptor (Figure 5A and B) [24,50,73]. Previous study identified that PHPS1 inhibit PI3K/AKT pathway with estrogen treatment, and here we found that inhibition of SHP2 in the presence of EGF, FGF2 or LIF reduced the expression of the PI3K/p-AKT genes expression (Figure 2E, Figure 4A, Figure 5B,E) [74]. Absence or inhibition of signaling of growth factors highly effect embryo development by activating apoptosis and also reducing embryo cell number [75]. Our results resemble to some extent with previous study that SHP2 inactivation in embryo (in the absence of growth factor) or SHP2 knockout from embryo highly enhance apoptosis and reduce embryo cell number (Figure 3B,C) [50].

Cytokines are not only important for invitro oocyte maturation, but it also play critical role in invitro oogenesis [69,76]. LIF is most frequently used cytokine and have significant effect in the invitro development of embryos of several species [26,69,77]. The relation between SHP2 and LIF receptor is well known, as SHP2 dephosphorylate and restrain STAT3 from nuclear localization [78,79,80]. Furthermore, previous studies demonstrated that cytokine activated JAK/STAT pathway is essential for blastocyst ICM development and SHP2 knockout also reduced the ICM [50,61]. In bovine embryos, we found a non-conventional relation between p-SHP2 and p-STAT3 for the development of ICM (Figure 5F). Inhibition of SHP2 phosphatase activity is directly proportional to p-STAT3 in the presence of LIF, as inhibition of SHP2 reduced and scattered p-STAT3 from ICM, but the exact phenomenon needs further studies.

SHP2 play an important role in stem cells, as SHP2 homozygous deletion from human and mouse embryonic stem cells failed both the ESCs to differentiate into germ layers [81]. Also, SHP2 conditional knockout mouse revealed that it plays key physiological role in male reproductive system and maintenance of spermatogonial stem cells [82]. We found that SHP2 inhibition highly reduced bovine blastocysts ICM (Figure 2D, Figure 5C) [50]. Defective ICM also reduced implantation potential of embryo, and previous study also identified that SHP2 knockout embryos showed defective trophoblast (Figure 2E) [50]. Furthermore, uterine specific SHP2 deletion completely restrain embryo’s invasion of maternal tissue [67]. So previous studies and also our study support that SHP2 expression is essential for embryo as well as for maternal uterine tissue for the successful implantation of embryo.

**Figure 6:**
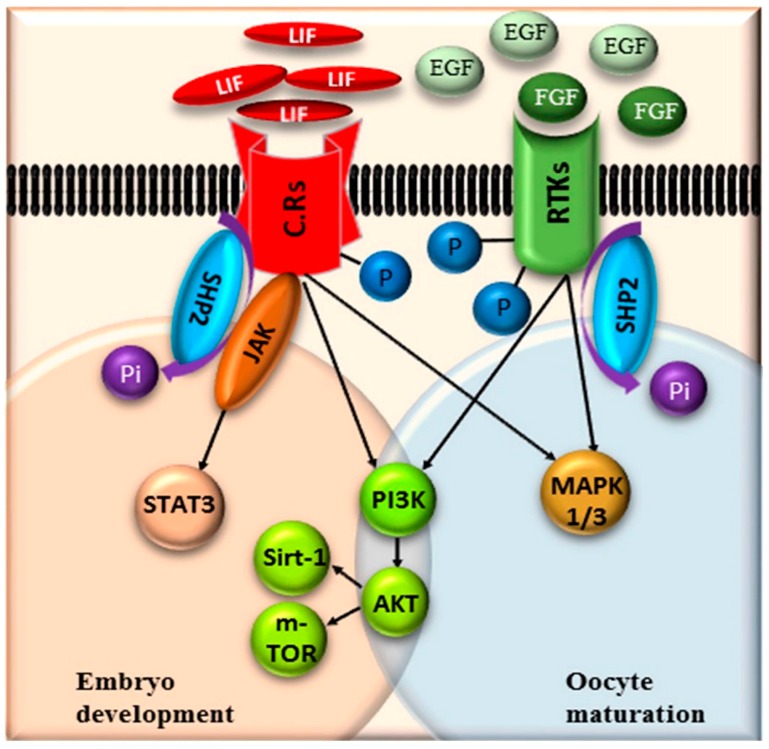
Schematic representation of the potential pathway for SHP2 de-phosphorylation of receptor tyrosine kinases (RTKs) and cytokine receptors (CR) in oocytes and blastocysts. Upon ligand binding these receptors are hyper-phosphorylated and SHP2 de-phosphorylate them by taking phosphate group, as a result the adaptor proteins get attached to the receptors and signal transduction starts.

Cisplatin a selective activator of SHP2, was previously used as chemotherapeutic drug for ovarian cancer [36]. Stephanie Morgan et al., stated that cisplatin at high concentration (5 μg) enhances premature ovarian failure (POF), but imatinib (inhibitor of tyrosine kinase signaling) neutralizes the anti-apoptotic effect of cisplatin [55,83]. In contrast to the previous studies our results suggest that EGF (tyrosine kinase signaling activators) not only neutralizes cisplatin apoptotic effect, but also enhances downstream signaling of RTKs (Figure 3 and Figure 4) [84]. Highly activated RTKs and cytokines signaling improve oocyte maturation and significantly enhance embryo development (Figure 6).

## 5. Conclusions

In conclusion, this study revealed for the first time SHP2 expression in bovine theca cells of pre-ovulatory follicles and in cumulus cells of COCs. SHP2 activation/inhibition significantly influence bovine oocyte maturation and embryo development. Furthermore, the phosphatase activity of SHP2 is important for oocyte maturation and embryo development, and also for the growth factors and cytokines signal transduction. It is also noteworthy to mention that SHP2 is in direct proportion with p-STAT3 during bovine embryo ICM development. 

## Figures and Tables

**Table 1:** Primers, their accession number, sequence and size used in qRT-PCR.

Name	Accession No.	Order Name	Sequence (5′-3′)	ProductSize (bp)
**PTPN11**	XM_002694590.6	F	GGCACAGTACTACAACTCAA	100
		R	TGGTCTCAGCTAATTTGCTT	
**MAPK1**	NM_175793	F	CCGTGTTGCAGATCCAGAC	130
		R	GACGGACCAGATGTCGATG	
**MAPK14**	NM_001102	F	GCTGTCGACCTGCTGGAGAAGATG	110
		R	TCGTCGTCAGGATCGTGGTACTGG	
**CPEB1**	XM_864691	F	GTGTGGAGTGGCCTGGTAAG	114
		R	GAGAGCAAGCCTGAAGCAAG	
**MAPK8**	NM_001192974.1	F	GACGCTTGATTGCATGTAAA	153
		R	TACCTCAAAGGGCTTCATTC	
**mTOR**	XM_002694043.6	F	TTAACAGGGTTCGAGACAAG	114
		R	AGAGGTTTTCATGGGATGTC	
**PI3K**	NM_174574.1 234	F	TCAACCATGACTGTGTGCCA	234
		R	CCATCAGCATCAAATTGGGCA	
**AKT3**	NM_001191309.1	F	AGCTGTTTTTCCATTTGTCG	94
		R	TGTAGATAGTCCAAGGCAGA	
**SIRT1**	NM_001192980.2	F	CAACGGTTTCCATTCGTGTG	138
		R	GTTCGAGGATCTGTGCCAAT	
**BAX**	NM_173894	F	CACCAAGAAGCTGAGCGAGTGT	118
		R	TCGGAAAAAGACCTCTCGGGGA	
**BCL-2**	NM_001166486.1	F	TGGATGACCGAGTACCTGAA	123
		R	GAGACAGCCAGGAGAAATCAAA	
**SOX2**	NM_001105463	F	CTATGACCAGCT CGCAGA	152
		R	GGAAGAAGAGGTAACCACG	
**STAT3**	NM_001012671	F	CTCTCCCCACTTCTGCCAAG	118
		R	AGGGGTCACAACTGCTGCTC	
**GAPDH**	NM_001034034	F	CCCAGAATATCATCCCTGCT	185
		R	CTGCTTCACCACCTTCTTGA	

Abbreviations: F, forward; R, reverse.

**Table 2:** Cleavage and development percentage of bovine embryos, Control verses PHPS1 (SHP2 inhibitor).

Groups	No. of Presumed Zygotes	No. of Cleavage Embryo (% ± SEM)	No. of Blastocysts (% ± SEM)
**Control**	339	254 (75.71 ± 2.76) ^a^	84 (31.86 ± 1.16) ^a^
**PHPS1**	316	154 (50.00 ± 6.44) ^b^	47 (16.14 ± 2.53) ^b^

^a,b^ Values with different superscripts in the same column are significantly different (*p* < 0.05). This experiment was completed in 7 replicates.

**Table 3:** Cleavage and developmental rates of embryos generated from oocytes with various media compositions.

Groups	No. of Presumed Zygotes	No. of Cleavage Embryos (% ± SEM)	No. of Blastocysts (% ± SEM)
**SOF**	789	475 (68.25 ± 2.14) ^a^	175 (20.25 ± 0.86) ^a^
**COMBO**	748	633 (84.00 ± 1.45) ^b^	294 (41.00 ± 0.94) ^c^
**CISPLATIN**	770	480 (66.25 ± 1.33) ^a^	149 (18.00 ± 1.00) ^a^
**CONTROL**	769	599 (79.75 ± 1.63) ^b^	240 (31.25 ± 0.56) ^b^

^a,b,c^ Values with different superscripts in the same column are significantly different (*p* < 0.05). This experiment was completed in 15 replicates.

**Table 4:** Leukemia inhibitory factor and fibroblast growth factor 2 cleavage percentage reversed by PHPS1.

Groups	No. of Presumed Zygotes	No. of Cleavage Embryos (% ± SEM)	No. of Blastocysts (% ± SEM)
**SOF + FGF-2**	456	335 (74.88 ± 3.34) ^a^	255 (32.22 ± 1.66) ^a^
**SOF + FGF-2 + PHPS1**	432	211 (48.58 ± 3.64) ^c^	154 (15.00 ± 1.03) ^b^
**SOF + EGF + LIF**	452	369 (81.78 ± 1.54) ^a^	259 (33.33 ± 2.88) ^a^
**SOF + EGF + LIF + PHPS1**	448	257 (57.89 ± 1.89) ^b^	171 (13.67 ± 1.59) ^b^

^a,b^ Values with different superscripts in the same column are significantly different (*p* < 0.01). This experiment was completed in 9 replicates.

## Supplementary Materials

The following are available online at https://www.mdpi.com/2073-4409/8/10/1272/s1, **Figure S1**: Expression of SHP2 in GV and MII oocytes, and the dose dependent effects of PHPS1 and Cisplatin; **Figure S2**: Effects of PHPS1 on ROS generation, developmental arrest and ICM damage; **Figure S3**: The effects of SHP2 expression on GVBD induction and fertilization.
