# Supplementary material for: PTPN11 (SHP2) Is Indispensable for Growth Factors and Cytokine Signal Transduction During Bovine Oocyte Maturation and Blastocyst Development
Source: Cells. 2019 Oct 18;8(10):1272. doi: 10.3390/cells8101272 (PMC6830097; doi:10.3390/cells8101272)
Supplement: Supplementary file 1 [file cells-08-01272-s001.pdf]

Supplementary data

Supplementary Figure 1

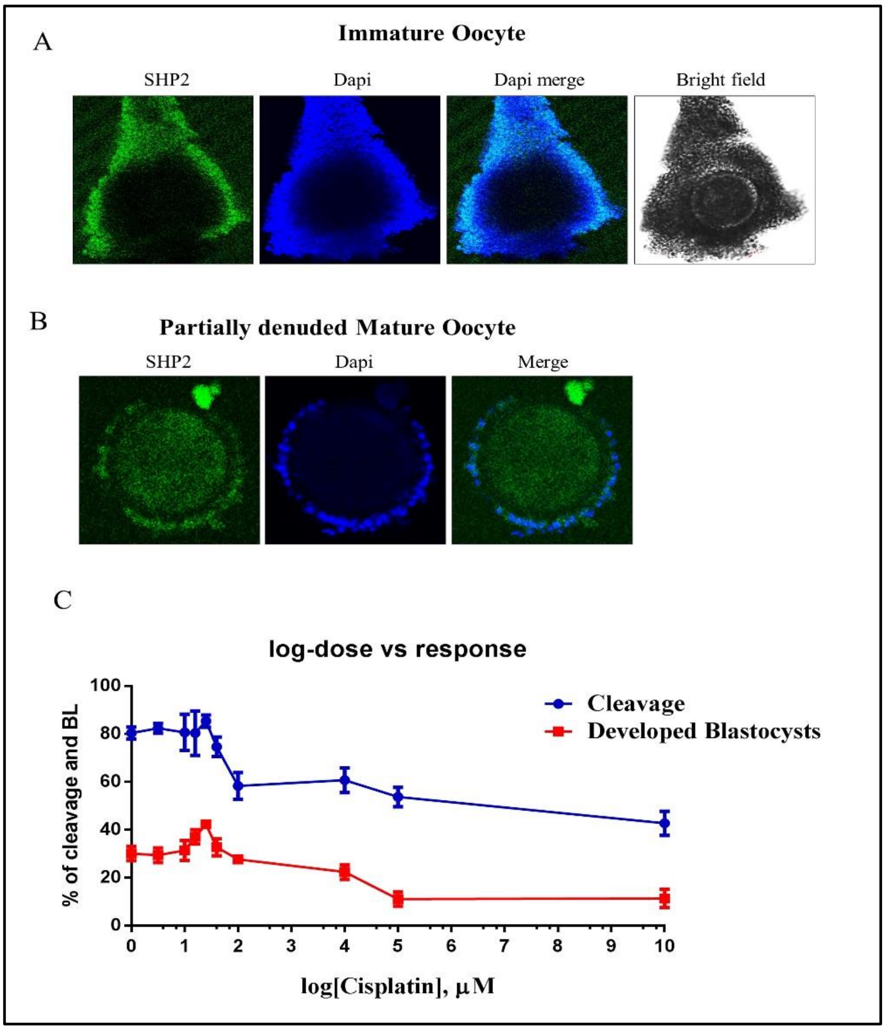

Figure S1. Expression of SHP2 in GV and MII oocytes, and the dose dependent effects of PHPS1 and Cisplatin. (A) Immunofluorescent staining of SHP2 in COCs (GV) showing the nuclear localization of SHP2 expression in cumulus cells (n=20 oocytes per each group). (B) Immunofluorescent image of SHP2 in partially denuded matured oocyte showing SHP2 expression in oocyte as well as in surrounding cumulus cells (n=20 oocytes per each group). (C) Log-dose dependent response curve of Cisplatin showing percentage (%) of cleaved and developed blastocysts (n=140-160 oocytes for each dose). The experiments were repeated 3 times and the data are shown here as a mean ± S.E.M. *P , 0.05, **P , 0.01, ***P , 0.001.

Supplementary Figure 2

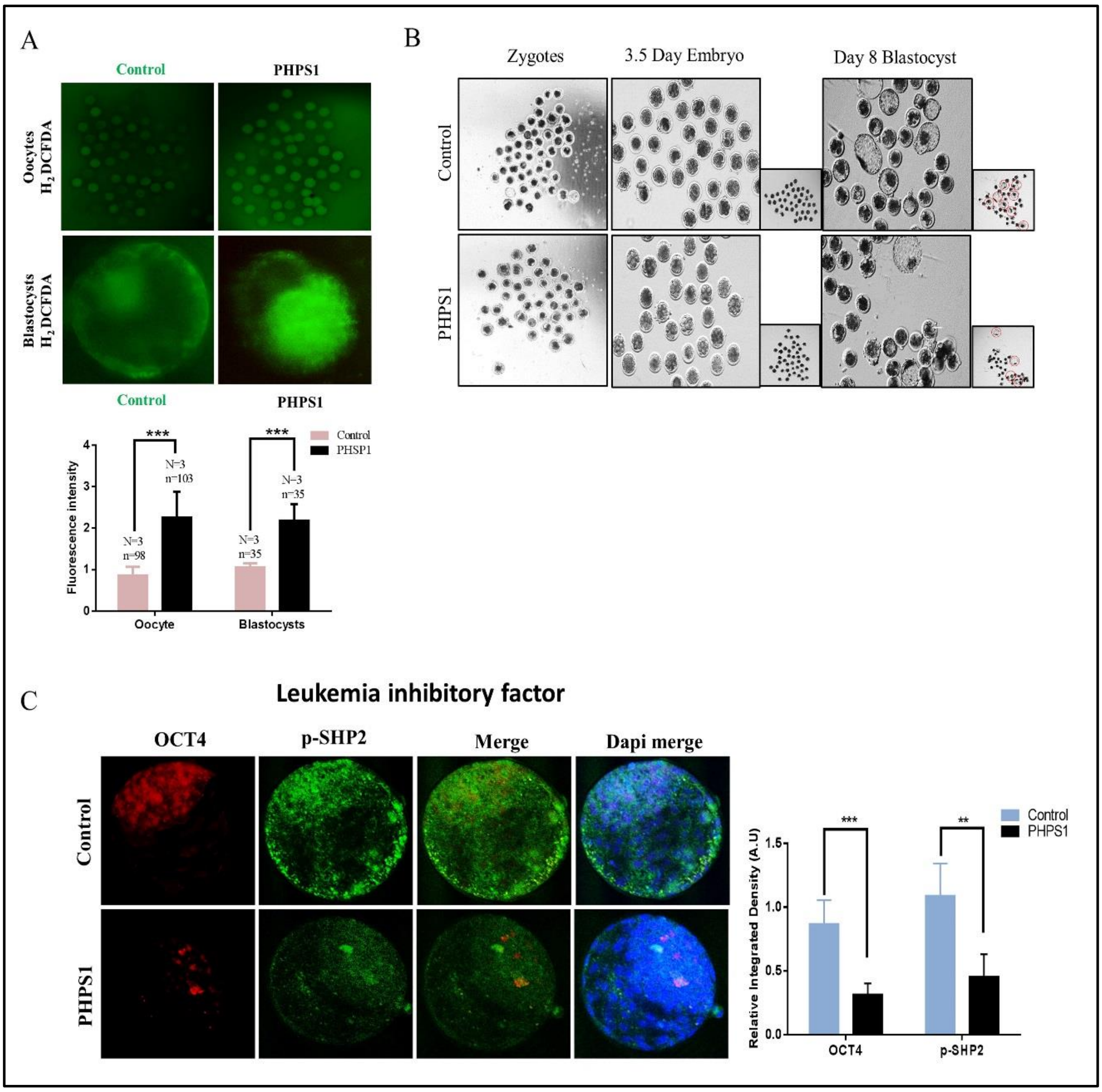

Figure S2. Effects of PHPS1 on ROS generation, developmental arrest and ICM damage. (A) H2DCFDA staining for ROS (reactive oxygen species) detection in oocyte and in blastocysts. (B) Embryo cleavage and blastocysts development in the control vehicle-treated and in PHPS1-treated group (Table 2). (C) Immunofluorescent images of OCT4 and SHP2 co-staining in the LIF and PHPS1-treated groups (n=20). The experiments were repeated 3 times and the data are shown here as a mean ± S.E.M. $^{**}P$, 0.01, $^{***}P$, 0.001.

Supplementary Figure 3

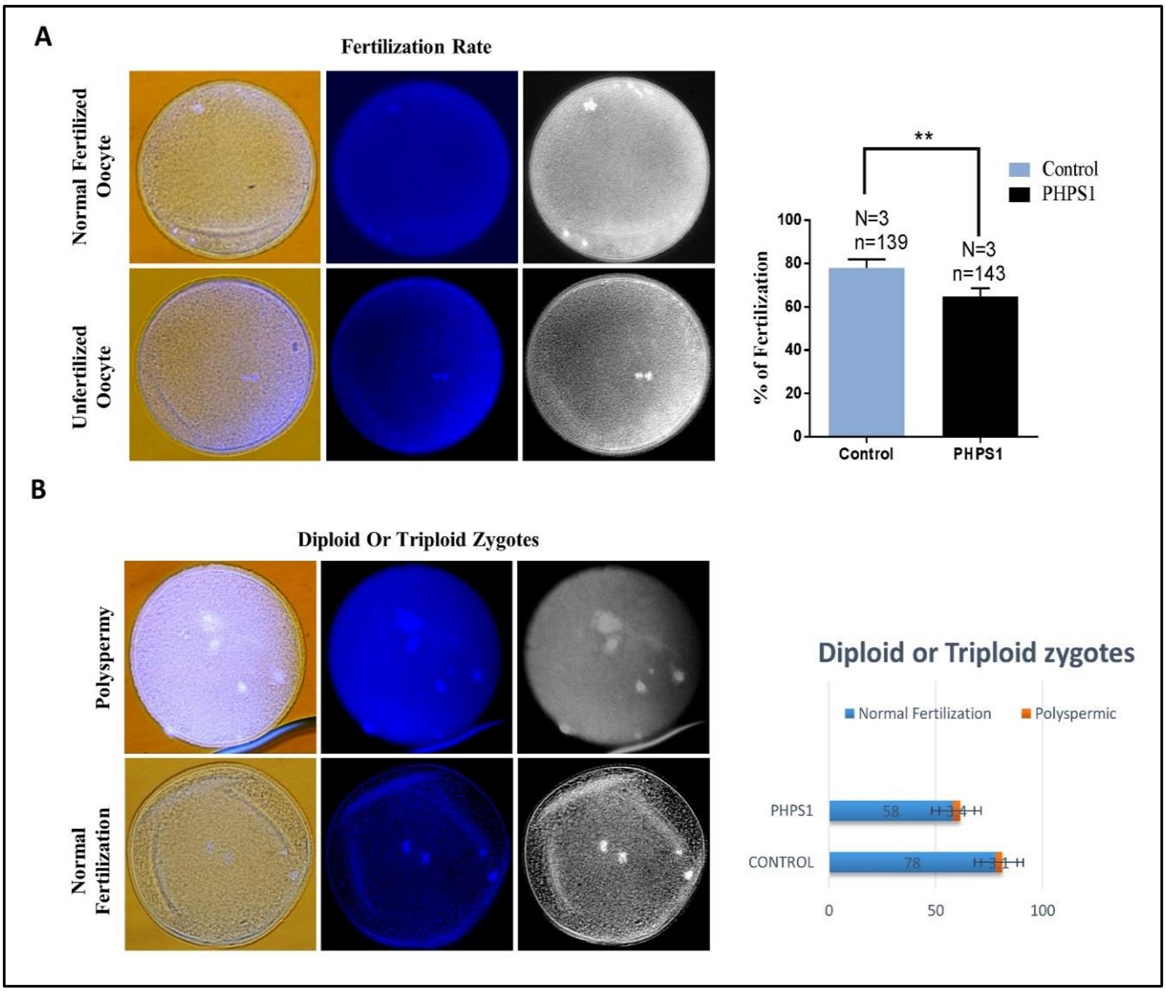

Figure S3. The effects of SHP2 expression on GVBD induction and fertilization. (A) SHP2 inhibition with PHPS1 significantly reduced fertilization. Independent experimental repeat (N) and used oocyte (n) values are as indicated. (B) The number of Polyspermic zygote were non-significant as compare to normally fertilized zygotes in both experimental groups (n=20 oocytes per each group). The experiments were repeated 3 times and the data are shown here as a mean ± S.E.M. N.S, not significant. **P* , 0.05, ***P* , 0.01.
